# Polish Version of the Post-traumatic Stress Disorder Related to COVID-19 Questionnaire COVID-19-PTSD

**DOI:** 10.3389/fpsyt.2022.868191

**Published:** 2022-04-25

**Authors:** Justyna Kosydar-Bochenek, Sabina Krupa, Francesca Favieri, Giuseppe Forte, Wioletta Medrzycka-Dabrowska

**Affiliations:** ^1^Department of Emergency, Institute of Health Sciences, College of Medical Sciences of the University of Rzeszow, Rzeszow, Poland; ^2^Department of Psychology, Sapienza University of Rome, Rome, Italy; ^3^Body and Action Lab, Istituto di Ricovero e Cura a Carattere Scientifico (IRCCS) Fondazione Santa Lucia, Rome, Italy; ^4^Department of Anaesthesiology Nursing and Intensive Care, Faculty of Health Sciences, Medical University of Gdansk, Gdańsk, Poland

**Keywords:** COVID-19, pandemic, post-traumatic stress disorder, PTSD, healthcare workers, self-report questionnaire

## Abstract

**Objective:**

Translate and investigate psychometric properties of the Polish version of COVID-19-PTSD in a sample of healthcare workers.

**Methods:**

The PTSD symptoms were investigated among 184 participants (physicians, nurses, and paramedics). The respondents completed Post-Traumatic Stress Disorder Related to COVID-19 Questionnaire (COVID-19-PTSD) via online survey. The psychometric properties (i.e., internal consistency, validity, and reliability) of the Polish version of COVID-19-PTSD were analyzed.

**Results:**

The findings showed that the Polish version of COVID-19-PTSD is a reliable instrument. The total and subscale scores demonstrated good internal consistency. We also found that the prevalence of PTSD was reported at around 32% of healthcare workers.

**Discussion:**

The Post-Traumatic Stress Disorder Related to COVID-19 Questionnaire (COVID-19-PTSD) is a first tool designed to assess the severity of PTSD symptoms related to the pandemic. The findings of our study confirmed good validity and reliability of the Polish version of COVID-19-PTSD which can be recommended to be used as a reliable screening tool to conduct psychological screening among Polish healthcare workers.

## Introduction

Post-traumatic stress disorder (PTSD) refers to a mental health condition occurring in an individual who experienced/witnessed a terrifying or traumatic event that is beyond the limit of personal psychological endurance. PTSD may result in significant psychological distress, cognitive dysfunction, and impairment in social and occupational areas of functioning (1). The four core symptoms of PTSD include (i) recurrent thoughts and feelings concerning the traumatic experience, (ii) constant avoidance of stimuli related to the traumatic event, (iii) negative changes in cognition and mood, and (iv) sustained increased alertness (2).

The outbreak of a pandemic, with no vaccines or any effective medical therapy, such as COVID-19, could be described as a traumatic experience due to its acute and chronic implications at both individual and community levels (3, 4). Being a major viral outbreak in the 21st century, the COVID-19 pandemic has resulted in an extraordinary burden on mental health worldwide (5, 6). Proportionately elevated rates of symptoms of anxiety (6.33–50.9%), depression (14.6–48.3%), post-traumatic stress disorder (7–53.8%), psychological distress (34.43–38%), and stress (8.1–81.9%) are observed in the general population at the times of the first outbreak of COVID-19 pandemic in China, Spain, Italy, Iran, the US, Turkey, Nepal, and Denmark (5, 7–11).

Moreover, the pandemic experience has called attention to its consequences on mental health among healthcare workers involved in the first-line response. Frontline health workers are experiencing severe stress and anxiety, facing increased workloads, and are being confronted with great suffering and high mortality rates (12). They are often forced to make difficult decisions that can cause ethical dilemmas with traumatic consequences. Their stress is aggravated by the risk of infection and transmission to the family. Unfortunately, there have been reports of social stigmatization of people working with people with COVID-19, while what they need is everyone's support. These experiences can have long-term emotional and functional consequences (13). Numerous studies demonstrated that a great share of healthcare workers is at significant risk for developing PTSD and Posttraumatic Stress Symptoms (PTSS) (14). Therefore, adequate mental health counseling for medical staff and other healthcare workers involved is a crucial task for public health (15, 16).

Escalation of PTSD during the pandemic requires a reliable assessment tool allowing to evaluate this condition. The present study investigates the Post-Traumatic Stress Disorder Related to COVID-19 Questionnaire (COVID-19-PTSD) - a 19-item self-report measure assessing the presence and severity of PTSD symptoms. From a clinical perspective the COVID-19 pandemic may be considered as a traumatic event. Therefore, psychological support to mitigate short- and long-term psychopathological consequences of the COVID-19 pandemic seem to be necessary (4). This study aimed to translate and investigate psychometric properties of the Polish version of COVID-19-PTSD in a sample of healthcare workers.

## Methods

### Study Design

A prospective descriptive study was conducted.

### Post-traumatic Stress Disorder Related to COVID-19 Questionnaire

A new self-report questionnaire (COVID-19-PTSD) was developed based on the PTSD Checklist for DSM-5 (PCL-5) questionnaire and administered to investigate its psychometric properties. The respondents are instructed as follows: “Referring to the current situation, characterized by the COVID-19 outbreak and the social distancing measures implemented to contain it, indicate how you feel for each of the following dimensions” (4).

The COVID-19-PTSD questionnaire includes 19 items, requiring a response on a 5-point Likert scale, from 0 (not at all) to 4 (extremely). To calculate the scale scores, add the items. A COVID-19-PTSD score of 26 was deemed to correctly categorize a participant as having or not having significant PTSD symptoms (4).

The psychometric properties of COVID-19-PTSD have been thoroughly investigated among the Italian population. The studies proved satisfactory psychometric properties of the COVID-19-PTSD questionnaire. Moreover, excellent internal consistency of all items was observed (Cronbach's α = 0.94). Cronbach's alpha for every item of the subscales was good in terms of the DSM-5 four-factors model (Cronbach's α = 0.70–0.86), as well as the seven-factors model (Cronbach's α = 0.52–0.85) (2). Confirmatory factor analyses results for Monofactorial Model, DSM-5 4-factors model and 7-factors model is shown in Table 1. COVID-19-PTSD questionnaire showed a significant correlation with the Impact of Event Scale—Revised (IES—R) (*r* = 0.70, *p* < 0.0001) which indicates good convergent validity. Moreover, significant positive correlations were found for all the IES—R subscales (ranging from 0.39 to 0.66) (4).

**Table 1:** Confirmatory factor analyses results for Monofactorial Model, DSM-5 4-factors model and 7-factors model.

**Fit indices**	**Monofactorial model**	**DSM-5 4-factor model**	**7-factor model**
X2/df	29.31^*^	25.65^*^	14.45^*^
CFI^a^	0.84	0.86	0.93
TLI^b^	0.82	0.84	0.91
RMSEA (CI 95%)^c^	0.11 (0.108–0.114)	0.10 (0.101–0.107)	0.07 (0.072–0.079)
SRMR^d^	0.06	0.05	0.06

a *Comparative Fit Index (cut-off ≥ 0.90)*.

b *Tucker-Lewis Index (cut-off ≥ 0.90)*.

c *Root Mean Square Error of Approximation (cut-off <0.08)*.

d *Standardized Root Mean Square (cut-off ≤ 0.08)*.

* *p <0.0001*.

According to the authors of the original questionnaire, COVID-19-PTSD is the first tool assessing PTSD in a situation of prolonged stress resulting from a pandemic (4). Therefore, it might be suitable to investigate its properties in other populations and professional groups.

### Setting and Procedure

The present study was conducted in two phases. The first phase was translation and cultural adaptation of the Italian version of COVID-19-PTSD into Polish. The second phase was the validation (face validity, content of COVID-19-PTSD). Consent was obtained from authors of original scale, who agreed and they also watch over the development of the Polish version. This study was conducted between May 1st and July 31th, 2021 that is 14 months from recording the first case of COVID-19 in Poland.

### The Translation Process

Before implementing the study in Poland, it was necessary to translate the scale and culturally adapt to Polish conditions. The authors of the original version of the scale were informed about the translation and expressed consent for the translation into Polish.

### Phase I. Translation and Cultural Adaptation

#### Forward Translation

Two native translators fluent in Italian and Polish individually translated the Italian version of the instrument into Polish.

#### Reconciliation

Two versions of the translation were reviewed and discussed item by item to achieve consensus regarding the best possible translation. The authors compared the two translations, and the final version was prepared after applying a few changes.

#### Back Translation

The preliminary version was then translated back into Italian by an experienced and certified language teacher without knowledge of the original version.

#### Back Translation Review

The back translation of the preliminary Polish version was then thoroughly compared with the original text regarding the necessity of performing adjustments. This back translation showed no substantial deviations from the original after close comparison and assessment performed by the translating authors. The translation was done by an independent translator specializing in medical translation, who accepted the version sent by the research team.

#### Pre-testing and Cognitive Interviewing

In order to examine the final version, 10 healthcare workers and researchers employed simultaneously at a University and a clinical hospital were randomly selected as critical judges. The people sent us their opinions about the difficulty, irrelevancy, and ambiguity of each item (qualitative face validity).

#### Final Version

After combining some minor revisions, the final Polish version of the instrument was developed.

### Phase II. Validation of the Polish Version of COVID-19-PTSD

Convenience sampling was used in this study. Participants were recruited from the clinical hospital and one of the temporary COVID-19 hospitals established by the Polish government. Physicians, nurses, and paramedics with a high risk of infection and psychological stress were invited to participate in the study. We chose an online questionnaire to survey because face-to-face survey was impractical given the requirement of quarantine and risk of viral transmission from close personal interaction. Besides, the online survey was fast, easy and convenient for data collection and analysis. Participants were selected from those hospital departments that involved either direct contact care to suspected or confirmed 2019-nCoV cases. This included the emergency department, outpatient clinic, infectious disease, and intensive care unit. One selection criterion was that the current working environment is at high risk of infection, i.e., reported close contact with COVID-19 patients or pathogens. A total of 250 subjects were successfully recruited in this study. The questionnaire was sent to employees via e-mail with the management consent. A brief presentation informed the participants about the aims of the study, and electronic informed consent was required from each participant before starting the investigation. At the beginning participants were required to fill in a short demographic questionnaire, and to respond to questions about personal experience related to the COVID-19 outbreak. Then, the questionnaire was administered. To guarantee anonymity, no personal data, which enabled the identification of the respondents, was required. Due to the aim of the current study, the only inclusion criterion was to be at least 18 years of age and work as a doctor, a nurse, or a paramedic in the healthcare system. The completion of all questionnaires took about 10–15 min.

### Statistics

Descriptive analysis was used to characterize the study sample in terms of demographic information. For reliability, the internal consistency of COVID-19-PTSD was accessed using Cronbach's alpha coefficient, where 0.70 was considered satisfactory. In order to examine the influence of sociodemographic variables on the occurrence of PTSD, the chi-square test (χ^2^) was used to analyze the distribution of PTSD incidence in sociodemographic groups. The level of significance was at *p* < 0.05. Student's *t*-test was used to compare the mean levels of stress/anxiety/risk associated with the possibility of COVID-19 infection in the groups with and without PTSD. The level of significance was at *p* < 0.05. Analyses were performed using Statistica 13.3.

## Results

### Participant Demographic Information and COVID-19-PTSD Score

The link to the survey has been sent to 250 healthcare workers. A total of 189 respondents participated in the study. Of the total respondents that started the questionnaires, 97% (184 out of 189 people) completed the whole survey and were considered for the statistical analyses. There were 131 women (71% of the sample) and 53 men. The mean age of the participants was 34.32 (SD = 14.43), and the age ranged between 24 and 49 years. Most of the participants completed higher education at BSc level (56%), and 38% graduated from MSc studies (Table 2).

**Table 2:** Participants' characteristics (*n* = 184).

**Characteristic**		***n***	**%**
Gender	Female	131	71
	Male	53	29
Age (years)	24–34	98	54
	35–45	78	42
	>46	8	4
Educational level	Post-secondary school	12	6
	Bachelor's degree	103	56
	Master's degree or more	69	38
Medical department	Emergency	73	40
	Intensive care unit	52	28
	Infectious disease	35	19
	Outpatient clinic	24	13
Psychopathological history	Yes	23	13
	No	151	82
Quarantine Experience	Yes	149	81
	No	35	19
Infection by the SARS-CoV-2	Yes	74	40
	No	110	60
Knowledge of people who died of COVID-19	Yes	129	70
	No	55	30

PTSD symptoms were evaluated according to the DSM-5-criteria. In the current study, we applied the 4-factors model which classifies PTSD symptoms into four specific criteria: Re-experiencing, Avoidance, Negative alterations in cognition and mood, and Increased arousal and reactivity. We also examined a seven-factors structure (Intrusion, Avoidance, Negative Affect, Anhedonia, Dysphoric arousal, Anxious arousal, and Externalizing behavior), and a A monofactorial structure of PTSD, according to the first version of COVID-19-PTSD.

Participants reported an average sum score of 17.47 (SD = 15.99) on the COVID-19-PTSD. A COVID-19-PTSD score of 26 was deemed to correctly categorize a participant as having or not having significant PTSD symptoms. The prevalence of PTSD, considering this cut-off score, was reported at around 32%. Means, standard deviations, minimum and maximum values for total score of COVID-19-PTSD and its subscales are presented in Table 3.

**Table 3:** Normative data for the COVID-19-PTSD and subscales.

**Scale**	**M**	**SD**	**Me**	**Min**	**Max**
COVID-19-PTSD	17.47	15.99	12	0	54
**4-factors model**					
Re-experiencing	3.24	4.06	1	0	16
Avoidance	1.52	1.92	1	0	8
Negative alterations in cognition and mood	5.70	5.23	4	0	19
Increased arousal and reactivity	7.01	6.68	5	0	22
**7-factors model**					
Intrusion	3.24	4.06	1	0	16
Avoidance	1.52	1.92	1	0	8
Negative affect	2.11	2.62	1	0	9
Anhedonia	3.59	3.46	3	0	12
Dysphoric arousal	1.64	1.89	1	0	6
Anxious arousal	1.92	2.18	1	0	7
Externalizing behavior	3.45	3.43	3	0	12

*M, medium; SD, standard deviations*.

As a result of the analysis, more frequent occurrences of PTSD stress was found in women than in men (81 vr 19%; χ^2^ 4.19; *p* = 0.04). The highest intensity of PTSD symptoms was observed in the group of emergency departments, while the lowest in staff of the outpatient clinic (29 vr 14%; χ^2^ 23.47; *p* < 0.001). Higher percentage of PTSD was observed in people without COVID-19 risk factors (86 vr 14%; χ^2^ 22.67; *p* < 0.001). The participants who were in quarantine had a higher total score of PTSD than healthcare workers who were not in quarantine (74 vr 26%; χ^2^ 12.36; *p* = 0.002). Those infected with SARS-CoV-2 had a higher PTSD percentage than the ones who were not infected (38 vr 62%; χ^2^ 9.54; *p* = 0.002). The subjects who knew someone who died of COVID-19 were significantly more likely to have a higher PTSD rate than those who did not know anyone who died of that disease (97 vr 3%; χ^2^ 35.57; *p* < 0.001). A statistically significant relationship was found in the case of the level of education - people with higher education have a higher intensity of PTSD symptoms than people with post-secondary education (*p* < 0.1).

In participants without PTSD symptoms, the subjective assessment of the level of stress/anxiety/threat related to the possibility of COVID-19 infection was on average 4.46 ± 2.41 (median scores: 5 on a Likert scale from 1 to 10).

**Figure 1:**
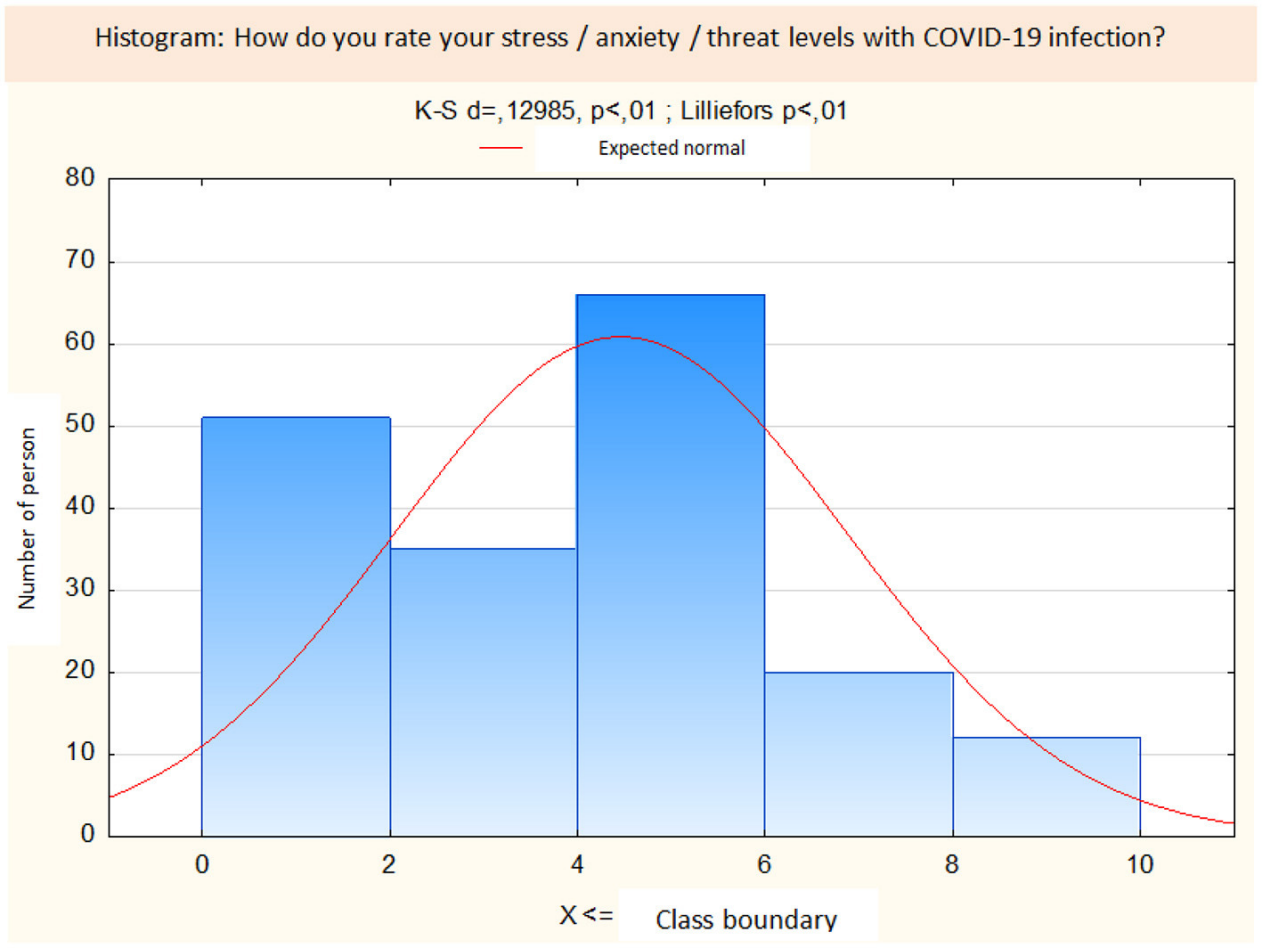
Distribution histogram of self-assessment of the level of stress/anxiety/risk related to the possibility of COVID-19 infection.

In people with PTSD, the assessment of the level of stress/anxiety/risk related to the possibility of COVID-19 infection was 6.67 ± 2.64 and was significantly higher than in the participants without PTSD and the average was 3.62 ± 1.76 (*t* = *8.02, p* < 0.0001). The results are presented in Figure 1.

### Psychometric Property of the Polish Version of COVID-19-PTSD

Cronbach's alphas were calculated for the internal consistency of COVID-19-PTSD. The Cronbach's coefficients of subscale scores in terms of the seven-factor model and DSM-5 four-factor model were summarized in Table 4. The Cronbach's alpha coefficient of the total score was 0.94, higher than the threshold of 0.70 and indicates high reliability of COVID-19-PTSD Polish version. Internal consistency reliability for each subscale is also satisfactory. The subscale results in the four-factor structure with good internal consistency (reliability ranging from 0.79 to 0.92). The mean correlation of the subscale scores with the total was ranging from 0.46 to 0.75. Also, the 7-factor structure evidenced good internal consistency (reliability from 0.72 to 0.92). The mean correlation of the subscale results with the whole was from 0.46 to 0.75. Based on the collected data, the Spearmann-Brown Coefficient was calculated, which is 0.85.

**Table 4:** Reliability analysis of the COVID-19-PTSD.

	**Cronbach's alpha**	**Mean correlation**
**4-factor model**		
Re-experiencing	0.92	0.75
Avoidance	0.79	0.66
Negative alterations in cognition and mood	0.82	0.46
Increased arousal and reactivity	0.88	0.54
**7-factor model**		
Intrusion	0.92	0.75
Avoidance	0.79	0.66
Negative Affect	0.72	0.47
Anhedonia	0.88	0.73
Dysphoric arousal	0.79	0.66
Anxious arousal	0.76	0.62
Externalizing behavior	0.86	0.71

## Discussion

The present study aimed to test the reliability and validity of the Polish version of COVID-19-PTSD to investigate COVID-19 related trauma during COVID-19 pandemic among healthcare workers. We demonstrated that the Polish version of COVID-19-PTSD had good psychometric indices (i.e., internal consistency and validity). The internal consistency of the total (Cronbach's alpha = 0.94) and subscale scores in the context of seven-factor structures (alpha = 0.72–0.92) was comparable to original version of the scale (alpha for total score = 0.94, and for subscale score in terms of four-factor structure = 0.52–0.85) (4).

Another finding of the present study was that a high percentage of healthcare professionals (32%) reported PTSD symptoms (average sum score of 17.47). These results are in line with previous studies reporting a high percentage of PTSD symptomatology (29.5%) and ower psychological wellbeing related to COVID-19 diffusion in the Italian population (4, 9).

Our results using the PCL-5 scale to investigate PTSD linked to COVID also highlight that healthcare workers at pandemic frontline are at higher-risk becoming infected and experiencing negative psychological outcomes including PTSD and burnout, anxiety, fear of transmitting infection, feeling of incompatibility, depression, increased substance-dependence (17, 18). Moreover, mental issues linked to the health emergency (i.e., PTSD, anxiety, depression, and sleep disorders) are more often observed in healthcare workers, mainly those at pandemic frontline (19). Accordingly, increase of PTSD symptoms from moderate to severe among the healthcare worker was showed in different worldwide hospital, such as China (20, 21), Saudi Arabian (22), Italian (23), Spanish (24–26). Generally, the percentage of PTSD symptoms ranging from 10 to 40% of healthcare workers. Elevated post-traumatic symptomatology correlated with age below 50 and <10 years of work seniority (24). Higher incidence was found among frontline workers than non-frontline ones (21).

Moreover, moderate to severe symptoms of psychological distress were observed in the healthcare professionals: depression (21%), anxiety (20%) and PTSD (29%), associated with burnout, prior psychiatric history, profession and resilience, in spite of low levels of COVID contact (25, 26).

A recent meta-analysis on psychological effect of outbreaks such as SARS, MERS, COVID-19, ebola, and influenza A on doctors, nurses, and auxiliary staff indicated that from 11 to 73.4% of them had PTSD symptoms during epidemic/pandemic, while in 10–40% they lasted up to 3 years after the traumatic event (27). These results are in line with other findings demonstrating that 37.8% healthcare staff during SARS/MERS/COVID-19 complained of psychological distress (95%CI = 28.4–48.2%, *k* = 15, *n* = 24,346) while 20.7% of PTSD (95%CI = 13.2–31%, *k* = 11, *n* = 3,826) (25). Accordingly, it could be interesting to develop longitudinal studies to follow the trend of PTSD symptoms among the healthcare population.

The prevalence of PTSD in the study group seems to be high compared to other studies, which may be due to various reasons. The data was collected during the peak period of infection and death from COVID-19 in Poland, characterized by a large overload of the national health care system and the restrictive measures adopted by the government, which could be perceived as a very acute stressor. In addition, the respondents were asked to focus only on COVID-19 issues, which could have influenced the perception of the pandemic as a highly traumatic event.

In sum, healthcare staff are particularly in danger of developing adverse mental health outcomes due to coronavirus. However, character and prevalence of these outcomes still need to be investigated (28). Therefore, monitoring mental wellbeing of healthcare workers as well as providing psychological support are warranted at times of COVID-19 pandemic (25, 29).

## Study Limitations

The limitations of this study are as follows: First, as this is the first study investigating psychometric properties of COVID-19-PTSD in the adapted version, no similar study about validation is available in any language to compare cross-cultural similarities and differences in reliability and validity of the scale. Since the original COVID-19-PTSD was developed in Italy, it has been validated only in the Italian population so far, therefore, discussion was a challenge. However, the specific sample adopted for the validation of the Polish COVID-19-PTSD generated interesting insight in the analysis of the phenomenon in a particularly vulnerable population. The results of our study in the discussion are partially related to studies conducted with the use of the PCL-5 scale, which is the prototype of the COVID-19-PTSD scale. Secondly, since the sample size was small, the statistical strength of the study may be diminished. To obtain better generalizability, future studies on a larger population and other samples are needed. Finally, the data collection in the more chronic period of the pandemic (following the first year of pandemic spread) could be influenced the results of the analysis, although according to the previous literature the evidence may show the maintenance of critical thresholds of PTSD symptoms even in the period following the first and sudden outbreak.

## Conclusions

The results of the present study suggest that the Polish version of COVID-19-PTSD demonstrated acceptable reliability and validity in the sample of Polish healthcare workers. This tool is very important from a clinical and practical point of view. The COVID-19-PTSD could be considered a sensitive screening tool in a population faced with pandemic, and it can represent a first step toward the assessment of the risk of PTSD. This instrument can be used to identify and reduce the risk of PTSD related to the COVID-19 pandemic. It may facilitate establishing and improving the warning mechanism of the PTSD crisis for early intervention of potential PTSD patients during and after 2019-nCoV disaster. Furthermore, COVID-19-PTSD is a time sparing tool due to its simplicity and small number of items. This is the first and comprehensive tool that is DSM-5 compliant and refers to the COVID-19 pandemic as a traumatic event. The COVID-19-PTSD questionnaire may also be useful for measuring symptom severity of PTSD in other populations. For better generalizability, future studies on a larger population and other samples are needed. Focusing on the risk of PTSD during the COVID pandemic, may lead to the development of effective prevention programs and therapeutic interventions. The impact of COVID-19 on the physical and mental health of healthcare workers should become a priority of the global public health strategies. Comprehensive Understanding PTSD is possible if further research is conducted. It is important to undertake research on different groups. Thanks to the from pathogenesis to intervention approach, it is possible to create a program that reduces PTSD in healthcare workers.

## Data Availability Statement

The original contributions presented in the study are included in the article/supplementary material, further inquiries can be directed to the corresponding author/s.

## Ethics Statement

The studies involving human participants were reviewed and approved by Bioethics Committee of the University of Rzeszow (KBE No. 13/11/2020). The patients/participants provided their written informed consent to participate in this study.

## Author Contributions

JK-B: conceptualization and resources. FF: formal analysis. JK-B and SK: methodology. WM-D: supervision. SK, GF, and WM-D: writing—original draft. All authors contributed to the article and approved the submitted version.

## Conflict of Interest

The authors declare that the research was conducted in the absence of any commercial or financial relationships that could be construed as a potential conflict of interest.

## Publisher's Note

All claims expressed in this article are solely those of the authors and do not necessarily represent those of their affiliated organizations, or those of the publisher, the editors and the reviewers. Any product that may be evaluated in this article, or claim that may be made by its manufacturer, is not guaranteed or endorsed by the publisher.
